# Serum Vitamin A and Inflammatory Markers in Individuals with and without Chronic Obstructive Pulmonary Disease

**DOI:** 10.1155/2015/862086

**Published:** 2015-08-03

**Authors:** L. M. O. Caram, R. A. F. Amaral, R. Ferrari, S. E. Tanni, C. R. Correa, S. A. R. Paiva, I. Godoy

**Affiliations:** ^1^Department of Internal Medicine, Pneumology Area, Botucatu Medical School, Universidade Estadual Paulista (UNESP), Botucatu Campus, Distrito de Rubião Junior, s/n, 18618-970 Botucatu, SP, Brazil; ^2^Department of Pathology, Botucatu Medical School, Universidade Estadual Paulista (UNESP), Botucatu Campus, Distrito de Rubião Junior, s/n, 18618-970 Botucatu, SP, Brazil

## Abstract

*Background.* Vitamin A is essential for the preservation and integrity of the lung epithelium and exerts anti-inflammatory effects.* Objective.* Evaluating vitamin A in the serum and sputum and testing its correlation with inflammatory markers in individuals with or without COPD.* Methods.* We evaluated dietary intake, serum and sputum vitamin A, tumor necrosis factor alpha, interleukin- (IL-) 6, IL-8, and C-reactive protein in 50 COPD patients (age = 64.0 ± 8.8 y; FEV_1_ (forced expiratory volume in the first second) (%) = 49.8 ± 16.8) and 50 controls (age = 48.5 ± 7.4 y; FEV_1_ (%) = 110.0 ± 15.7).* Results.* COPD exhibited lower serum vitamin A (1.8 (1.2–2.1) versus 2.1 (1.8–2.4) *μ*mol/L, *P* < 0.001) and lower vitamin A intake (636.9 (339.6–1349.6) versus 918.0 (592.1–1654.6) RAE, *P* = 0.05) when compared with controls. Sputum concentration of vitamin A was not different between groups. Sputum vitamin A and neutrophils were negatively correlated (*R* ^2^ = −0.26; *P* = 0.03). Smoking (0.197, *P* = 0.042) exhibited positive association with serum vitamin A. COPD was associated with lower serum concentrations of vitamin A without relationship with the systemic inflammation. *Conclusions.* Serum concentration of vitamin A is negatively associated with the presence of COPD and positively associated with smoking status. Sputum retinol is quantifiable and is negatively influenced by neutrophils. Although COPD patients exhibited increased inflammation it was not associated with serum retinol.

## 1. Introduction

Vitamin A is essential for the preservation of the integrity of the epithelium, and it exerts anti-inflammatory effects in the lungs. Vitamin A deficiency promotes and aggravates preexisting inflammation [1, 2]. Previous studies have demonstrated that the risk for chronic obstructive pulmonary disease (COPD) increased with decreasing levels of serum vitamin A [3, 4]. Furthermore, cross-sectional studies have reported that the intake and serum concentration of vitamin A are associated with the degree of airway obstruction in smokers and COPD patients, and the serum concentration of vitamin A is lower in patients with moderate or severe COPD than in nonsmoking controls [5, 6]. In addition, improvement in pulmonary function is achieved with vitamin A supplementation [5].

COPD is associated with chronic inflammation, and there is increasing evidence that systemic inflammatory mediators, such as C-reactive protein (CRP), tumor necrosis factor alpha (TNF-*α*), interleukin- (IL-) 8, and IL-6, are elevated in the peripheral blood of COPD patients [7, 8]. Some studies have demonstrated an association between systemic inflammation and a decrease in serum vitamin A concentrations [9–11]. Possible contributing mechanisms for the decrease in vitamin A during the course of inflammatory diseases include the decreased synthesis of retinol-binding protein by the liver and increased vascular permeability at sites of inflammation, thereby allowing leakage of retinol-binding protein to the extravascular space and the loss of the vitamin in the urine [12]. Another mechanism proposed to explain the decrease in the serum concentration of vitamin A is the increased demand to repair the damage caused by acute or chronic inflammation [12]. We hypothesized that vitamin A concentration measured in the serum may be related to increased levels of inflammatory markers and may be associated with the presence of COPD. Thus, the aim of the present study was to evaluate vitamin A levels in the serum and sputum and to test whether the concentration of vitamin A is correlated with markers of inflammation in the peripheral blood of individuals with or without COPD. In addition, we measured, for the first time, vitamin A levels in induced sputum and tested whether the level of sputum vitamin A was associated with local markers of inflammation.

## 2. Material and Methods

We performed a cross-sectional study at the Botucatu Medical School, located in the city of Botucatu, Brazil, evaluating 50 clinically stable patients with COPD (GOLD II: 20 (40%), GOLD III: 12 (24%), and GOLD IV: 18 (36%)) and 50 controls (61% current smokers). COPD was diagnosed according to the criteria established by the Global Initiative for Chronic Obstructive Lung Disease [13]: a forced expiratory volume in 1 s/forced vital capacity (FEV_1_/FVC) ratio <70% after the administration of a bronchodilator (400 *μ*g of fenoterol) without significant reversibility (<11% predicted FEV_1_ or 200 mL). All COPD patients were lifelong smokers (smoking history > 20 pack-years), and 39% were active smokers. The following exclusion criteria were applied: oral steroid use or COPD exacerbation in the last three months before enrollment in the study; diagnosis of another chronic or respiratory disease; and inability to understand the study protocol. All controls underwent routine clinical assessment, including spirometry and a chest X-ray. The Research Ethics Committee of the Botucatu Medical School approved the study design, and all participants provided written informed consent.

### 2.1. Pulmonary Function Tests and Oximetry

We determined the forced expiratory volume in the first second (FEV_1_) and the forced vital capacity (FVC) based on the flow-volume curve obtained using a spirometer (Koko; Ferraris Respiratory, Louisville, CO) before and 20 min after inhalation of a beta 2-agonist (fenoterol, 400 *μ*g). The highest value of at least three measurements, expressed as percentages of reference values, was selected [14]. Oxygen saturation (SpO_2_%) was evaluated using a portable oximeter (Nonin Medical, Plymouth, MN).

### 2.2. Nutritional Assessment

The nutritional assessment included the measurement of height and weight, and body mass index (BMI) was calculated as (kg/m^2^). The typical daily nutrient intake during the past six months was estimated using a food-frequency questionnaire [15]. The instrument listed 120 food and beverage items. Using the computer software NutWin, Nutrition Program (Information Health Science Center of São Paulo Federal University, São Paulo, Brazil, 2002), we converted dietary information into energy, protein, fat, and vitamin A intake values (retinol activity equivalent, RAE).

### 2.3. Blood Sampling and Sputum Induction

Fasting peripheral blood samples were collected in the early morning (between 8:00 and 10:00 a.m.), and the serum was stored at −80°C until analysis. We followed the European Respiratory Society recommendations for the induction and processing of sputum [16, 17] as previously described [18], and all procedures were conducted under red light.

### 2.4. Determination of Serum and Sputum Retinol Concentration

We used reverse-phase HPLC to measure retinol concentrations in the serum and induced sputum [19]. Aliquots of the serum and sputum supernatant were prepared for extraction [19] and assayed using a C18 column (Symmetry C18, 46 × 75 mm; 3.5 *μ*m). The HPLC system consisted of a separation module (Waters Alliance 2695; Waters, Milford, MA) with a photodiode array detector (2996; Waters), which we set to 325 nm. The HPLC mobile phase was water-acetonitrile-tetrahydrofuran (30 : 50 : 20, by vol., with 1% ammonium acetate in water; solvent A) and water-acetonitrile-tetrahydrofuran (6 : 50 : 44, by vol., with 1% ammonium acetate in water; solvent B). The gradient procedure, at a 1 mL/min flow rate (16°C), was as follows: 2 min in 85% solvent A and 15% solvent B; 9–19 min in 17% solvent A and 83% solvent B; a 1min hold in 100% solvent B; and 21–30 min in 85% solvent A and 15% solvent B. We quantified the retinol concentration by determining peak areas in the HPLC chromatograms calibrated against known standard quantities. We corrected for extraction and handling losses by monitoring recovery of the internal standard.

### 2.5. Quantification of Inflammatory Mediators in the Serum and Supernatant of Induced Sputum

Were assessed tumor necrosis factor alpha (TNF-*α*), interleukin- (IL-) 6, and IL-8 levels in duplicate using high-sensitivity commercial enzyme-linked immunosorbent assay kits, according to the manufacturer's instructions (BioSource International Inc., Camarillo, CA). The lower detection limit was 0.09 pg/mL for TNF-*α*, 0.16 pg/mL for IL-6, and 0.39 pg/mL for IL-8. We assessed serum C-reactive protein (CRP) levels, also in duplicate, using a high-sensitivity particle-enhanced immunonephelometry (CardioPhase; Dade Behring Marburg GmbH, Marburg, Germany) with a lower detection limit of 0.007 mg/L.

### 2.6. Statistical Analyses

The mean ± SD or the median interquartile range (25–75%) was used to present the results according to the data distribution. The subjects were separated into two groups based on the diagnoses of COPD (absence or presence). When comparing the two study groups, an unpaired *t*-test was used for continuous variables, and the Mann-Whitney *U*-test was used for ordinal variables. The chi-square test or Fisher's exact test was used to evaluate the qualitative variables. For robust multiple linear regressions, clinically relevant variables were selected. The included categorical variables were sex (female = 0, male = 1), presence of COPD (absence = 0, presence = 1), interaction of IL-6 × CRP × TNF-*α* (was considered by multiplying the variables to construct one variable), and age, as a continuous variable.

All the data were analyzed using the software SigmaStat 3.2 (SPSS Inc., Chicago, IL, USA) and STATA. Statistical significance was defined as *P* < 0.05.

## 3. Results

In this study, 100 subjects were included in the analysis of the results. Sputum samples were obtained from COPD patients (*n* = 50) and smoker controls (*n* = 19).  Table 1 presents the general characteristics of individuals grouped according to diagnoses (controls and COPD). Gender and active smoking status did not differ statistically between the groups. Patients with COPD exhibited lower values of spirometric variables, functional capacity, and fat-free mass (Table 1).

The analyses of inflammatory markers revealed higher serum concentrations of TNF-*α* (*P* = 0.05), IL-6 (*P* < 0.001), CRP (*P* < 0.001), neutrophils (*P* < 0.001), and leukocytes (*P* < 0.001) in COPD patients when compared with controls. IL-8 levels and lymphocytes did not exhibit a statistically significant difference between the groups (Table 2). In sputum, TNF-*α* (8.18 (1.53–33.98) versus 1.10 (0.38–6.86) pg/mL, *P* = 0.01) was higher in COPD patients than in smoker controls. Sputum IL-6, IL-8, neutrophils, and leukocytes did not differ between the groups. The airway and systemic inflammation (TNF-*α*, IL-6, IL-8, and CRP) in COPD patients according to GOLD stage were not different between groups (data not shown).

**Figure 1:**
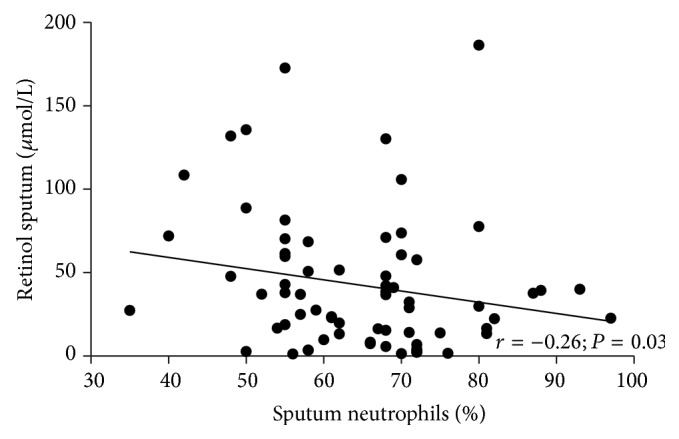
Correlation between retinol sputum and sputum neutrophil concentrations.

Table 3 presents the intake of protein, energy, and vitamin A and the concentration of vitamin A in the serum. COPD patients exhibited lower levels of protein ingestion in g/day (*P* = 0.01) and in g/Kg/day (*P* = 0.01) when compared with the controls. Furthermore, the vitamin A intake (*P* = 0.05) and the serum concentration of vitamin A (*P* < 0.001) were lower in the group with airway obstruction. The sputum concentration of vitamin A did not exhibit statistically significant differences (34.5 (8.1–57.6) versus 28.8 (18.0–66.6) *μ*mol/L, *P* = 0.38) between groups. However, vitamin A and neutrophils were negatively correlated in the sputum (*R* ^2^ = −0.26; *P* = 0.03) (Figure 1).

Age, gender, smoking status, and the presence of COPD were included as independent variables in robust multiple linear regression to identify factors associated with vitamin A serum concentrations (Table 4). Smoking status (0.197, *P* = 0.042) exhibited a positive association with vitamin A concentrations, and the presence of COPD was associated with lower concentrations of vitamin A (−0.480, *P* = 0.001) (Table 4).

## 4. Discussion 

The present study demonstrated that COPD patients exhibited lower intake and serum concentration of vitamin A when compared with controls. Furthermore, active smoking is positively associated with the serum concentration of vitamin A. Although our data confirm previous findings demonstrating that COPD patients exhibit higher concentrations of TNF-*α*, IL-6, CRP, neutrophils, and leukocytes when compared with controls, the serum concentration of vitamin A was not associated with inflammatory markers. The study also showed for the first time that vitamin A is quantifiable and is negatively influenced by the percentage of neutrophils in induced sputum.

Lower serum concentrations of vitamin A among COPD patients when compared with controls and a significantly negative influence of COPD diagnoses on vitamin A concentrations have been previously described [3–5, 20]. Paiva et al. (1996) and Lin et al. (2010) demonstrated that the serum concentration of vitamin A was lower in patients with COPD than in controls [5, 21]. In addition, McKeever et al. (2008), using data from the Third National Health and Nutrition Examination Survey, demonstrated that higher serum levels of antioxidant vitamins (vitamin A) were independently associated with higher levels of FEV_1_ [6].

The physiopathology for the lower serum concentrations of vitamin A in COPD remains unclear. Our results revealed that vitamin A intake was lower in COPD patients when compared with controls, which could explain, at least in part, the lower serum concentration. Our data are consistent with the data of Lin et al. (2010), who reported that vitamin A intake (4053 ± 2447 versus 5988 ± 3451, *P* = 0.009 (calculated from diet/1000 kcal total energy)) was lower in COPD patients than in controls [21].

Some studies have demonstrated an association between systemic inflammation and a decrease in serum vitamin A concentrations [9–11]. Our results demonstrated that COPD patients exhibited higher concentrations of inflammatory markers (TNF-*α*, IL-6, CRP, neutrophils, and leukocytes) compared with controls, which are in agreement with previous findings [22–24]. No influence of airway obstruction severity on airway or systemic inflammation in COPD patients was observed in our study. Previous findings also showed no difference in inflammatory markers between COPD patients at different GOLD stages [25]. Analyzing data from the Third National Health and Nutrition Examination Survey, Stephensen and Gildengorin demonstrated that chronic pulmonary disease was associated with elevated serum CRP concentrations and that serum retinol was lower in subjects with elevated CRP concentrations [9]. In contrast, in our study, vitamin A concentrations were not associated with inflammatory markers. This may be because the inflammation in COPD patients seems to be of low grade and is not persistent in the majority of the patients [23, 26]. Godoy et al. (1996) followed up weight losers (WL) and weight stable (WS) COPD patients for 6 months. The authors reported that TNF-alpha concentrations were significantly higher in the WL COPD patients when compared with WS patients at baseline. However, this difference was not maintained after 6 months of follow-up [26]. Furthermore, a recent study evaluated the systemic inflammatory state (white blood cells (WBC) count and CRP, IL-6, IL-8, fibrinogen, and TNF-*α*) in 1,755 COPD patients, 297 smokers with normal spirometry and 202 nonsmoker controls, who were followed up for three years. According to Agustí et al. (2012), at baseline, 30% of COPD patients did not exhibit evidence of systemic inflammation, and only 16% exhibited persistent systemic inflammation [23].

We have showed a positive association between smoking status and serum vitamin A concentrations and that retinol in induced sputum is quantifiable and is negatively influenced by the percentage of neutrophils. Therefore, our data obtained in sputum confirm that inflammation may have a negative influence on the concentration of retinol in the airways [9, 10]. We also showed that retinol in induced sputum did not correlate with vitamin A intake and the concentration of retinol in the serum. In agreement with our findings Redlich et al. (1996) evaluated retinol in bronchoalveolar lavage (BAL) cells and the lung tissue of 21 patients with respiratory disease and demonstrated that retinol was detectable in the BAL cells but exhibited no relationship with tissue or dietary/serum concentrations. However, the retinol concentration in the BAL cells was the best predictor of vitamin A concentrations in the lung tissue [27]. The concentration of vitamin A on lung tissue was not performed in our study; however, the data obtained in the sputum may indicate a local relationship between inflammation and lower levels of vitamin A on lung tissue.

Higher concentrations of circulating vitamin A in smokers have not been reported in previous studies [28–30]. In fact, a study evaluating 12,741 volunteers (7,713 women, 35–60 years of age, and 5028 men, 50–60 years of age) showed that smoking had no effect on differences in serum retinol between nonsmokers, former smokers, and current smokers (*P* = 0.987) [29]. We evaluated only the serum concentrations and not the reserves of vitamin A and it is possible that the vitamin in the lung tissues has been liberated to the systemic circulation in smokers. Therefore, this is a limitation of our study and does not permit a conclusion about the influence of smoking on the vitamin A status. Higher levels of systemic inflammation (IL-6 and CRP) in COPD than in smokers found in our study and in the Eclipse study [23] and lower intake of vitamin A in COPD compared to smokers (636.9 (339.6–1349.6) versus 918.0 (592.1–1654.6), *P* = 0.05) can explain why COPD patients and not smokers present decrease in serum vitamin A. There were other possible limitations to this study. The analyses were performed using cross-sectional data and therefore valid inferences regarding causal pathways cannot be drawn. For future investigations, larger study populations are needed.

## 5. Conclusions

In conclusion, our results demonstrate that low intake and serum levels of vitamin A are associated with the presence of COPD. The serum concentration of vitamin A is positively associated with smoking status. Although COPD patients exhibited increased inflammation, these inflammatory markers were not associated with serum retinol concentrations. Furthermore, sputum retinol is quantifiable and is negatively influenced by the presence of neutrophils. A new contribution of our study was that sputum measurements may be a tool to evaluate the nutritional status of the retinol in the airways.

## Figures and Tables

**Table 1:** General characteristics of individuals grouped according to diagnoses.

Variables	Controls (*n* = 50)	COPD (*n* = 50)	*P* value
Gender F/M (*n*)	21/29	20/30	0.80
Active smokers (%)	61	43	0.53
Pack/years	35.0 (26.2–41.5)	50.0 (30.0–60.0)	**0.01**
Age (years)	48.5 ± 7.4	64.0 ± 8.8	**<0.001**
FEV_1_ (%)	110.0 ± 15.7	49.8 ± 16.8	**<0.001**
FEV_1_/FVC (%)	81.0 (77.0–84.0)	48.0 (38.0–61.0)	**<0.001**
6 MWD (m)	562.5 (497.9–624.0)	411.0 (357.0–486.0)	**<0.001**
BMI (kg/m^2^)	24.4 (22.5–26.5)	24.8 (22.3–27.6)	0.41
FFM (kg)	47.0 (39.4–53.4)	42.1 (37.3–45.6)	**0.01**
IFFM (kg/m^2^)	17.4 (15.4–18.2)	16.3 (14.8–17.5)	0.06

Data are reported as the means ± SD or as the medians (interquartile range (25–75%)). F/M = female/male; FEV_1_ = forced expiratory volume in the first second (% of predicted); FVC = forced vital capacity (% of predicted); 6 MWD: six-minute walk distance; FFM: fat-free mass; IFFM: index fat-free mass. The *P* values refer to COPD compared with the control (unpaired *t*-test or Mann-Whitney test and chi-square).

**Table 2:** Inflammatory markers in the serum of control subjects and COPD patients.

Variables	Control (*n* = 50)	COPD (*n* = 50)	*P* value
TNF-*α* (pg/mL)	3.9 (3.6–5.4)	4.5 (4.1–5.1)	**0.05**
IL-6 (pg/mL)	0.3 (0.2–0.6)	1.1 (0.8–1.9)	**<0.001**
IL-8 (pg/mL)	4.9 (3.4–7.1)	4.1 (3.3–7.2)	0.48
CRP (mg/L)	1.1 (0.6–2.2)	6.3 (2.7–9.3)	**<0.001**
Neutrophils (cell/mm^3^)	3605 (2900–4342)	4712 (4037–5667)	**<0.001**
Lymphocytes (cell/mm^3^)	1750 (1529–2090)	1720 (1470–2397)	0.94
Leukocytes (cell/mm^3^)	6400 (5300–7300)	7600 (7000–9125)	**<0.001**

Data are reported as the median (interquartile range (25–75%)). TNF-*α*: tumor necrosis factor; IL-6: interleukin-6; IL-8: interleukin-8; CRP: C-reactive protein. The *P* values refer to COPD compared with the control (Mann-Whitney test and chi-square).

**Table 3:** Energy, protein, and vitamin A intake and serum vitamin A.

Variables	Control (*n* = 50)	COPD (*n* = 50)	*P* value
Energy (Kcal/day)	2545 (2034–2907)	2525 (1811–3092)	0.79
Energy (Kcal/kg/day)	39.4 (29.6–45.9)	36.7 (27.6–48.0)	0.73
Protein (g/day)	77.8 (64.7–102.1)	68.2 (43.5–89.4)	**0.01**
Protein (g/kg/day)	1.2 (0.9–1.5)	1.0 (0.7–1.4)	**0.01**
Vitamin A intake (RAE)	918.0 (592.1–1654.6)	636.9 (339.6–1349.6)	**0.05**
Vitamin A (serum) (*μ*mol/L)	2.1 (1.8–2.4)	1.8 (1.2–2.1)	**<0.001**

RAE: retinol activity equivalent. Data are reported as the median (interquartile range (25–75%)). The *P* values refer to COPD compared with the control (Mann-Whitney test).

**Table 4:** Robust multiple linear regression to identify factors associated with vitamin A concentrations.

Vitamin A (serum)	Dependent variables	Standardized coefficients	*P* value
	Age (years)	0.068	0.620
	Gender (male)	−0.161	0.086
	Smoking status (yes)	0.197	**0.042**
	IL-6 × CRP × TNF-*α* (pg/mL)	0.112	0.247
	Presence of COPD (yes)	−0.480	**0.001**

*R* ^2^ = 0.26. For robust multiple linear regressions, clinically relevant variables were selected. The included categorical variables were gender (female = 0, male = 1), smoking status (absence = 0, presence = 1), presence of COPD (absence = 0, presence = 1), the interaction of IL-6 × CRP × TNF-*α* (was considered by multiplying the variables to construct one variable), and age as a continuous variable.
